# Perspectives on the academic mission in Quebec’s health and social services system: a qualitative study

**DOI:** 10.1108/JHOM-02-2025-0105

**Published:** 2025-08-19

**Authors:** Julie Lane, Sèverine Lanoue, Maxime Guillette, Marie Massuard, Esther McSween-Cadieux

**Affiliations:** Université de Sherbrooke, Sherbrooke, Canada; Université TELUQ, Montreal, Canada

**Keywords:** Qualitative study, Health system, Academic health center

## Abstract

**Purpose:**

Academic health centers play a central role in integrating research, education and specialized care within health systems. In Quebec (Canada), these centers operate within a unique designation process that defines their academic mission and functions. This project, supported by the Ministry of Health and Social Services, explores how the academic mission is perceived and defined by those involved in its implementation, highlighting key functions, challenges and opportunities for its evolution.

**Design/methodology/approach:**

A qualitative case study was conducted in 2021, involving 43 group interviews with 322 participants from designated and non-designated health and social services centers, universities, public-sector health organizations and national institutes. Data were analyzed using content analysis, combining deductive and inductive approaches to identify key themes.

**Findings:**

The academic mission remains focused on supporting care and services to improve population health and well-being. Interviewees emphasized the need to create value from academic mission activities and strongly believe in their positive impact. However, they also identified multiple barriers, including lack of recognition, limited resources and integration challenges. While research and education functions were clearly defined, other functions – such as knowledge translation, diffusion, health intervention and technology assessment and leading practices – were more difficult to define due to uncertainties regarding their objectives, implementation and alignment within the academic mission.

**Originality/value:**

This study provides empirical insights into the evolving academic mission within an integrated health and social services system. It offers recommendations for refining and harmonizing the designation process and criteria and strengthening the academic mission’s impact.

## Background

### Academic health centers in the healthcare system

Academic Health Centers (AHCs) are present in the healthcare systems of most high-income countries (Edelman *et al.*, 2018). Recent literature reviews have explored the history, role, and mission of AHCs (Cardinaal *et al.*, 2021; Edelman *et al.*, 2018; French *et al.*, 2014; Gonzalo *et al.*, 2021; Raus *et al.*, 2020), consistently identifying three defining characteristics: (1) the pursuit of a tripartite mission, also called academic functions, which include specialized patient care and services, laboratory and clinical research, and the education of health professionals; (2) an affiliation between a university and a health center (or a between a university and a network of health centers); and (3) significant variability in the definitions, organizational structures, vision, and implementation of academic health missions, reflecting the complex nature of AHCs (Brown, 2018).

This variability has led several authors (Davies, 2009; French *et al.*, 2014; Sanfilippo, 2010) to emphasize that “*if you’ve seen one AHC, you’ve seen one AHC*” (Sanfilippo, 2010, p. 384), highlighting the unique characteristics of each institution. Surprisingly, few empirical studies have examined how AHCs define their vision and functions (Edelman *et al.*, 2018; French *et al.*, 2014; Raus *et al.*, 2020), with much of the existing literature consisting of editorials, viewpoints, or perspectives on specific AHC models, primarily from the United States. Current research also suggests that there is limited knowledge on how the academic mission is defined by those responsible for its implementation within healthcare systems.

Three types of AHC designation have been identified in the literature. Edelman *et al.* (2018) distinguished between self-designation, as seen in the United States (Institute of Medicine, 2004), and national designation, which is applied in countries such as the United Kingdom, Australia, and the Netherlands (Davies, 2009; Davies *et al.*, 2010; Edelman *et al.*, 2019). Additionally, some AHCs undergo specific accreditation processes in Taiwan and Iran, despite the absence of a national designation processes (Huang *et al.*, 2009; Safarani *et al.*, 2018).

### Context of the academic mission in Quebec’s health and social services system

In Canada, the healthcare system is publicly funded and primarily managed by provincial governments. In Quebec, the Ministry of Health and Social Services (MSSS [ministère de la Santé et des Services sociaux]) oversees health and social services, including institutions that hold an academic mission. Since 1991, the province has implemented a government-led designation model that formally recognizes institutions affiliated with a university and actively engaged in academic activities. This process regulates their academic mission based on criteria, reflecting the strategic role of these institutions in advancing care, research, and workforce development.

Quebec’s designation model is one of the earliest government-led approaches to regulating academic health institutions (Conseil médical du Québec, 1998), preceded similar initiatives in the Netherlands (1994) (Davies *et al.*, 2010), the United Kingdom (2009) (French *et al.*, 2014; Imperial College London, 2007) and Australia (2014) (Edelman *et al.*, 2019; NHMRC, 2021). The concept was introduced by the 1988 Rochon Commission and officially enacted through a revision of the Act Respecting Health Services and Social Services (LSSS, chapter S-4.2) in 1991 (Gouvernement du Québec, 2021a).

Three types of academic designations were established based on specific criteria, with the first granted in 1992 (Conseil médical du Québec, 1998). These correspond to distinct categories of university-affiliated institutions: (1) University Hospital Centers (centre hospitalier universitaire [CHU] – all designated in the healthcare sector), (2) University Institutes (institut universitaire [IU] - some designated in health and others in social services), and (3) Affiliated University Centers (centre affilié universitaire [CAU] – also spanning both sectors) (Conseil médical du Québec, 1998; Duplantie, 2005; Gouvernement du Québec, 2021a). Table 1 summarizes the characteristics of each type of designation. The academic functions and criteria associated with each designation may vary depending on the type, as each has distinct mandates and institutional contexts.

**Table 1:** Main characteristics of academic designations in Quebec

Designation type	University hospital centers (CHU)	University institutes (IU)	Affiliated university centers (CAU)
Field of expertise	Multiple fields (health)	One field (health or social)	One field (must complement an existing CHU or IU)
Research	Recognized center or institute	Recognized center or institute	Participate in research activities
Education	Health (mainly medicine)	Health or social (related to field)	Health or social (related to field)
Specialized care and services	Multiple specialized fields in health	More than one (linked to the field of expertise)	None required
Health interventions and technologies assessment (HITA)	In the fields of expertise	In the field of expertise	None required

**Source(s):** Authors’ own work

### Impacts of structural reforms on the academic mission

In 2004, the Quebec government implemented a major structural reform of the healthcare system, leading to the creation of Health and Social Service Centers (CSSS [*centre de santé et services sociaux]*). This reform integrated health and social service organizations under a single governance structure, merging local health and social service centers, long-term care facilities, and hospitals (Malouin, 2008; Wankah *et al.*, 2018). This integration required all institutions, regardless of whether their initial academic designation was in health or social services, to offer both types of services. While no comparable international models of designated academic centers in social services have been identified, several authors highlight the importance of integrating social responsibility and community engagement within AHCs (Borden *et al.*, 2015; Edelman *et al.*, 2018; Raus *et al.*, 2020; Sanfilippo, 2010; Smitherman *et al.*, 2019; Wartman and Steinberg, 2011).

**Figure 1:**
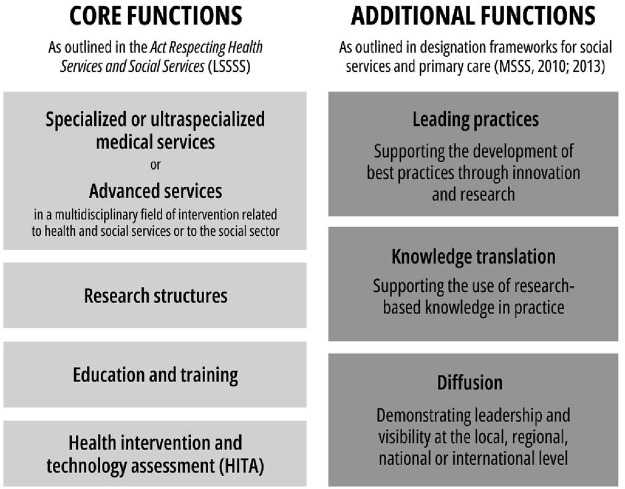
Functions guiding university designations in Quebec. Source: Authors’ own work

Since its inception, Quebec’s academic designation process has continued to evolve. A key distinguishing feature is the formal inclusion of social services alongside healthcare. As a result, academic designations have been granted not only in the health sector but also in the social services sector, each governed by its own set of criteria and requirements. The core academic functions associated with designation are embedded in legislation (specifically in articles 88 to 91 of the law- Loi sur les services de santé et les services sociaux [LSSSS]) and include four mandatory components: specialized care and services, research, education, and health intervention and technology assessment (HITA). Over time, other functions, such as knowledge translation (KT), diffusion and leading practices, have been added into official reference frameworks, particularly for social services designations. These functions serve as both eligibility criteria for designation and guiding pillars for how the academic mission is implemented in practice (Figure 1).

However, these developments have also exposed inconsistencies between sectors (Lane *et al.*, 2022), particularly in the designation criteria and the academic functions required. Moreover, only social services designations are subject to periodic renewal (every five years), whereas health designations remain indefinite. These discrepancies stem partly from the fact that health designations were established first and have never been revised.

These inequities were further exacerbated by a new structural reform introduced in 2015, which led to the creation of the Integrated Health and Social Services Centers (CISSS [*centre intégré de santé et services sociaux*]) (Gouvernement du Québec, 2021b). This reform merged CSSSs with other health and social services organizations, including rehabilitation centers, youth centers, and university hospital centers (CHU) (Wankah *et al.*, 2018). As a result, Quebec now has 34 large public-sector health and social services organizations, some of which cover vast geographical territories.

One outcome of this reform was the integration of multiple academic designations within newly merged, large-scale organizations. The newly formed Integrated University Health and Social Services Centers (CIUSSS [*centre intégré universitaire de santé et services sociaux*]) were not subject to a specific designation process. Instead, they were automatically granted the “university” label if at least one of their component facilities already held an academic designation in either health or social services (Gouvernement du Québec, 2021b). However, disparities emerged, with some CIUSSS containing only one designation while others house up to six. This new reality has intensified existing issues and highlighted conceptual ambiguities regarding the vision and functions of the academic mission, as institutions with different academic statuses were merged under a unified structure.

Over time, several challenges have emerged related to the academic mission: the multiplication of designation criteria to reflect institutional diversity; variation in the criteria and indicators used across designation types; a lack of harmonization in renewal procedures; and a wide range of terminology used to describe academic functions. These divergences have created confusion and hindered the development of a shared understanding of the academic mission and its functions. Considering these challenges, there is broad support for modernizing and clarifying the mission, aligning it with the integrated nature of today’s health and social service system. This study responds to that need by documenting how the academic mission is currently defined and enacted by those responsible for its implementation.

### Study objectives

This project aligns with the MSSS’s goal to harmonize and modernize the vision of the academic mission, ultimately supporting the implementation of a designation process with fair and adapted criteria for all AHCs. To assist the MSSS in the endeavor, our research team conducted a review of national and international literature alongside an extensive consultation with key stakeholders. This article aims to explore the perspectives of professionals involved in the academic mission, drawing on their experiences and perceptions to better understand their vision of the academic mission and the way academic functions are defined. Specifically, the study addresses the following key questions:

1. **Objective 1.** How should the academic mission be defined, and what are its objectives, impacts and modes of implementation?
2. **Objective 2.** What functions should constitute the academic mission, and how should they be defined and operationalized?

## Methods

### Study design and participant recruitment

To answer these research questions, we conducted a qualitative case study in 2021. This approach was chosen to gain an in-depth understanding of the academic mission by capturing the perspectives and lived experiences of participants (Creswell and Creswell, 2018; Yin, 2018). Group interviews were selected as the primary data collection method (Baribeau and Germain, 2010; Krueger and Casey, 2015), as they allow for rich discussions and exchanges among participants, facilitating the emergence of shared perspectives as well as contrasting viewpoints.

### Data collection

The group interviews lasted between 60 and 120 min and were conducted via Microsoft Teams between June and October 2021. They were audio-recorded for transcription and analysis. The lead investigators (JL, SL, MG, and MM) conducted semi-structured group interviews, following a predefined interview guide specifically developed for this study (see Supplementary file 1). The guide was structured around two central themes:

1. Defining the academic mission: What is its main purpose? What should be the outcomes? What are the barriers? How is it - or should it be - actualized?
2. Defining the functions of the academic mission: For each function, how does the function help the academic mission? What is this function purpose? How should this function be actualized and integrated?

This project was approved by the Research Ethics Committee – Education and Social Sciences, Université de Sherbrooke (reference number: 2021–3,220). All participants received both written and oral information and provided written informed consent before participating.

### Data analysis

Following data collection, group interviews recordings were transcribed by a neutral third-party professional. A “two voices” transcription approach was applied, distinguishing between the facilitator and the participants to ensure that individual participants could not be identified in the final transcripts. The data were analyzed using content analysis (Bardin, 2013) with the support of Nvivo software (version 12, 2018). A combined deductive and inductive approach was employed. The initial analysis, conducted by one team member (SL), categorized the data based on the main themes outlined in the interview guide, including the definition and vision of academic mission, the definition for each function, perceived impacts, actualization, and implementation. This preliminary analysis was then reviewed and validated by two team members (MG and MM). In the second phase, an inductive approach was applied to each major theme (SL, MG and MM), allowing key participants’ perceptions related to the research objectives to emerge. The final analyses were further refined and validated through multiple team discussions (SL, MG, MM, and JL) to ensure consistency and rigor.

## Results

A total of 43 group interviews were conducted, involving 322 professionals and managers engaged in the academic mission. Most participants (*n* = 203) were affiliated with both academic designated (*n* = 16) and non-designated (*n* = 8) health and social services centers. Additionally, group interviews were conducted with representatives from 15 other organizations involved in the academic mission and health and social services system, including universities, public-sector health organizations, national health research institutes, etc. Finally, interviews were also held with the executive boards of the four Integrated Academic Health and Social Services Regional Networks (RUISSS [*réseau universitaire intégré de santé et services sociaux*]). Table 2 provides a detailed overview of the participants’ organizations and their professional roles (for the participants from health and social services center).

**Table 2:** Participants’ organizations and roles

Roles and organizations	Frequency (n. Of participants)
*Health and social services centers*	
Higher management	34
General academic mission^a^	62
Care and services^b^	44
Specific academic designated area^c^	41
Administrative and support roles^d^	10
Other invited professionals^e^	12
*Other organizations involved in the academic mission*	
Government-related organizations and national institutes	34
Universities	36
Other public-sector health-related organizations	7
Executive boards of RUISSS	42
*Total*	*322*

**Note(s):**

a Designated and non-designated centers have managers responsible for the academic mission.

b Includes fields such as nursing; mental health; youth services; gerontology, etc.

c Following the 2015 reform, only specific areas within AHC are designated and have specific management.

d Includes roles in human resources; public health; quality and ethics, etc.

e Includes retired professionals, consultants, university representatives, etc.

**Source(s):** Authors’ own work

## Objective 1. defining a vision of the academic mission

### Purposes and perceived benefits of the academic mission

Participants widely agreed that the primary purpose of the academic mission is to meet the needs of the population and contributes to its health and well-being. This overarching goal implies that the academic mission:

[…] should be able to help us adapt and evolve our services according to the changing needs of the population and that […] all our actions should demonstrate that we can produce this result, which is to improve the situation of the population or the different populations we serve through the development of practices (group interview participant).

While the academic mission is more formally recognized in designated centers, participants emphasized that its ultimate purpose, which is to improve the health and well-being of the population, should extend beyond designated institutions:

I think there should be impacts on all other organizations. That is to say, when you have a university designation, you have a responsibility to bring something to the other organizations as well. (group interview participant)

The analysis revealed three key objectives related to the academic mission: (1) Excellence in the development, implementation, and integration of all academic functions within the broader health and social services mission; (2) Creation, development, sharing, mobilization, and application of scientific and experiential knowledge to enhance the quality of care and services across all levels and fields expertise and (3) Development of specialized expertise in priority areas of health and social services to address both current and future needs of the population and the health and social services system.

Aligned with these objectives, most participants emphasized that the academic mission should generate value for multiple stakeholders, including patients and their caregivers, the populations served, health and social services centers and their professionals, regional networks of health and social services and their partners, the MSSS, the government, and society at large. This broad scope of impact led many interviewees to identify numerous benefits associated with the academic mission. Table 3 presents examples of perceived benefits reported by participants.

**Table 3:** Perceived benefits of the academic mission in health and social services centers

Categories of benefits	Examples of benefits
Population (specially patients and caregivers)	improved quality of care and services Needs-based adaptation of care and services More integrated care and services
Health and social services personnel	Enhanced professional development (e.g., initial and continuing education, stimulating learning environment)
Health and social services centers and regional networks (CISSS, CIUSSS, RUISSS)	Development of learning health systems (learning organizations) Strengthened leadership and credibility National and international recognition and reputation Improved retention and recruitment of professionals Advancement of expertise and specialized knowledge Stronger partnerships enhancing services, research, educationetc.
Ministry, government and society	Scaling up of innovations in health and social services Influencing on national policies in health and social care Economic benefits through improved cost-effectiveness

**Source(s):** Authors’ own work

### Implementation and integration of the academic mission

Participants emphasized that components of the academic mission, particularly research and education, are present across all health and social services institutions, whether or not they hold a university designation. However, the scope and intensity of these activities, as well as the responsibilities that come with them, are greater in designated centers. To better support the academic mission’s implementation, several areas were identified: increasing its visibility and prioritization within organizations; developing an organizational culture that values continuous learning and knowledge sharing; allocating appropriate human and financial resources; creating internal governance structures adapted to institutional contexts; and strengthening collaboration both within and beyond the health and social services network.

However, participants identified several barriers and challenges. A major concern raised across all interviews was the lack of recognition for tasks associated with the academic mission. This issue is linked not only with the overall academic mission but with most of its core functions:

[…] if you want us to play a role in accompanying our colleagues, in co-development, in KT and participate in research projects, it must be valued […] (group interview participant)

As illustrated in the following statements, this recognition issue is closely linked to resource constraints, including human, administrative, time, and financial limitations:

[…] research and teaching should not be “on the side” but really be part of the culture of a health professional.

All the thoroughness that is required, but also the energy that is required in the development of a leading practice. It takes a lot of time not only for the team that accompanies but also for the clinical directors.

There are no dedicated funds, officially, that come with an academic designation. Everything must come from clinical budgets. It puts, in an institution, a competition between clinical and academic resources.

They [HITA units] struggle to deliver since they are always underserved in terms of resources for the number of questions they have to investigate

In addition to challenges related to implementation, integration emerged as another key issue raised by participants. While often difficult to achieve, integration was widely regarded as essential for fully realizing the academic mission. Participants emphasized the importance of aligning academic functions with one another, with clinical programs and services, and with broader organizational structures. This integration was described as a condition for creating synergy, breaking down silos, and enhancing the contribution of the academic mission to service improvement:

You can have academic functions, you can have them all, but when you are able to integrate them, you create a lot of synergy. (group interview participant).

Other participants highlighted the need for institutional integration, ensuring that the academic mission is embedded within the structure and culture of health organizations:

It is a priority the integration of the academic mission at all levels. (group interview participant).

Some interviewees also stressed the importance of regional-level integration, particularly through the RUISSS and cross-sector partnerships, to foster alignment across institutions and with population needs:

So for me, the integration zone, I think we need to strengthen the RUISSS […] (group interview participant).

### Importance of collaboration and partnership mechanisms

Participants frequently emphasized the importance of collaborating at different levels and with different partners, describing them as essential components despite the numerous barriers:

Everyone has goodwill, everyone wants to collaborate. But, without realizing it, we don’t always facilitate communication and collaboration, and that goes for our partners outside the organization as well as universities, which are our partners, other educational institutions, the ministries, and their agencies. We don’t have mechanisms to support collaboration. (group interview participant).

Despite these challenges, participants also shared many inspiring examples of successful collaboration initiatives. Collaboration occurs within health and social services centers for instance, between researchers and clinicians, and between centers, between designated and non-designated institutions for example. Various projects are conducted in partnership between centers and public-sector health organizations, national institutes, municipal and community organizations, and ministries. At the regional level, coordination efforts within RUISSS were mentioned as a promising avenue for strengthening the academic mission. The RUISSS structure is perceived as:

a place for concertation, knowledge development, knowledge sharing, and knowledge application. (group interview participant).

## Objective 2. defining the functions of the academic mission

Participants frequently defined the academic mission by referring to its various functions. Research and education were the most frequently mentioned, followed by KT. Less frequently cited were HITA and leading practices. Specialized and highly specialized care and services, as well as diffusion, were rarely discussed as core functions of the academic mission. Several interviewees described the academic mission in relation to these functions:

For me, the components of the “U” are obviously research and education. And these components must support the clinical mission

Basically, the notion of an academic mission in an institution is the recognition of different missions that are added to the mission of care. So, the education mission, the research mission, and then the health intervention and technology assessment

I think it really includes as much education, research, development of leading practices, innovations, and the element for me that’s really important because as I remember it’s kind of the desire to have an academic mission, is KT

Following these discussions, participants were asked to define each function and describe how it is, or should be, implemented within the academic mission.

### Research function

For most participants, research is considered a core component of the academic mission. There is a strong consensus that designated academic centers have a responsibility to advance knowledge and support the improvement of care and services, decision-making and policy development. However, opinions diverge regarding the focus of research. While some advocate for practice-changing research that directly influences clinical and organizational practices, others stress the importance of fundamental research, arguing that it should not be overlooked. This tension is highlighted in the following statement:

This is something that I constantly hear from managers, clinicians, and obviously from patients […] they often have this concern about “useful” research. And I put “useful” in quotation marks because if we define useful research, we must also define useless research (group interview participant).

Three main purposes were identified: (1) developing knowledge across all levels (from fundamental to clinical research), some of which contributes directly to the improvement of care and services (also referring to translational research); (2) generating specific knowledge based on the expressed needs of patients, populations, professionals, and leaders, to inform the provision of care and services as well as policy decisions; and (3) critically examining existing care and services to foster continuous improvement. The research function is carried out across both designated and non-designated centers, with varying levels of involvement, and in collaboration with multiple partners, including universities, national institutes, and other public and private organizations. Research is also viewed as a dynamic and interactive process, closely integrated with other academic functions, particularly in direct collaboration with HITA, using KT and diffusion, and in support of care, services, and leading practices.

Participants highlighted four areas that could help strengthen the implementation of the research function. First, they highlighted the need for stronger governance structures to foster alignment between clinical and research priorities, promote cross-sector collaboration, and facilitate knowledge co-construction between knowledge producers and users. Second, they called for a better balance between collaboration and competition, suggesting that all institutions, including non-designated ones, should have access to minimal support and be part of broader research networks. Third, the contribution of all organizations to research should be recognized, particularly the role of non-designated centers in enabling access to data and populations. Finally, participants emphasized the importance of valuing both applied and fundamental research, while acknowledging the operational challenges associated with conducting clinical research in practice settings.

### Education function

Education is recognized by interviewees as a key function of the academic mission, playing a fundamental role in both initial and continuous training for health and social services professionals. While the primary focus of this function seems to be on initial training, participants also highlighted its benefits for professionals already in practice:

At its core, this function is about training the next generation. […] It is also what students bring us, they come with a background, a knowledge that is up to date and related to best practices (group interview participant).

Participants identified five objectives of this function: (1) preparing students for their future careers by equipping them with the necessary skills and knowledge; (2) introducing new knowledge into institutions through students’ presence; (3) generating new learning opportunities for supervisors; (4) contributing to workforce development by aligning training with needs, and (5) promoting continuous learning for all employees.

While many interviewees noted that education is present in both designated and non-designated centers, they also emphasized the need to better recognize the contribution of non-designated institutions. Academic designated centers, however, were perceived as having specific responsibilities and capacities, including experimenting with innovative pedagogical approaches, developing specialized educational content aligned with their expertise areas and supervising students in research and ultra-specialized settings.

To strengthen this function, participants highlighted six priorities: positioning education more strategically at both ministerial and institutional levels; enhancing partnerships with educational institutions and professional bodies to better align training with workforce needs; allocating adequate resources; fostering a culture that values continuous learning and student integration; recognizing and supporting supervisors through training, formal roles, compensation and time release; and implementing student retention strategies, particularly in underserved regions.

### Specialized and highly specialized care and services function

Participants found it challenging to distinguish between the general mission of care and services and the specific role of specialized and highly specialized care and services within the academic mission. Some interviewees pointed out that excellence in care is not exclusive to designated centers, as high-quality specialized services can also be provided in non-designated centers. The key distinction of designated centers is that they focus on a specific area of expertise, where care and services are integrated into other academic functions. Data analysis led to the identification of a core aim for this function: to provide care and services in a specialized area of expertise, ensuring their development and integration across other academic functions. Most participants emphasized that research, education, HITA, leading practices, KT, and diffusion should be aligned with the designated area of expertise within the care and services function.

Participants raised concerns about the lack of coordination in how areas of expertise are recognized across institutions, which may result in duplication and fragmented efforts. While some emphasized the importance of maintaining institutional autonomy, others advocated for a more structured, system-wide approach to designating expertise areas—one that fosters complementarity, supports collaboration, and enables the strategic dissemination of innovations and best practices throughout the network.

### Health intervention and technology assessment (HITA) function

The HITA process begins with a request from a leader or senior manager seeking evidence-based information to support decision-making. The HITA unit then conducts a rigorous systematic review, integrating scientific, contextual and experiential evidence to inform these decisions. HITA is perceived as highly valuable, particularly among senior management:

HITA is extremely useful to support decisions. […] In my work, whenever possible, I will rely on scientific evidence, on best practices. When I come across a HITA report, I’m extremely happy because I know that I will have all that in the same document and that it’s going to help me support my position (group interview participant).

Based on participant insights, the HITA function serves two primary aims: (1) supporting decision-makers by providing evidence and best practices; and (2) guiding decision-makers in determining how evidence from HITA reports can be used and implemented in care and services. Despite its methodological rigor, the HITA function faces significant challenges, particularly regarding report production timelines, which are hindered by limited resources:

Often, we hear the famous classic HITA […] we are more in the 18–24 months of production. But the decision-maker, when he approaches us, needs an answer in the next few months (group interview participant)

To strengthen the HITA function, participants identified four areas: increasing the response capacity of HITA units, which are often under-resourced despite growing and time-sensitive demands; enhancing collaboration between units and across institutions to pool expertise, avoid duplication, and improve access to evidence products; diversifying methods and outputs (e.g. rapid reviews, abbreviated HITA, program evaluations, practice guides) to better align with decision-makers’ timelines and needs; and reinforcing implementation support to ensure recommendations are not only produced but translated into practice.

### The knowledge translation and diffusion functions

Participants found it difficult to distinguish between the KT function and the diffusion function within the academic mission. According to interviewees, KT is a proximity process that takes place within centers, between centers, and across health and social services networks, facilitating the exchange of knowledge between stakeholders. Diffusion refers to unidirectional dissemination of knowledge toward society, including the general population, and the international scientific community. While some participants associated both functions primarily with research, most emphasized that KT and diffusion must also be integrated with other academic functions, such as HITA, education, and leading practices.

Participants noted that KT serves four main objectives: (1) fostering dialog between researchers and practitioners; (2) providing access to, promoting, and influencing best practices in care and services based on relevant evidence-based knowledge; (3) supporting care and services units in achieving excellence and effectively responding to population needs, and (4) ensuring the continuous development of knowledge and skills among practitioners. The diffusion function aims to: (1) achieve international recognition for research and leading practices; (2) promote knowledge to a broad audience, and (3) support the definition and redefinition of public policies, ensuring that academic knowledge influences decision-making at various levels.

While many activities related to these functions are being carried out, interviewees perceived limited tangible impact. As one participant noted:

There is a multitude of KT activities across the network. We know that. But the problem is that there is a lack of follow-up on the use of this knowledge. It’s good to hold conferences, symposiums, and congresses, but afterward, what do we do with this knowledge? If we don’t know, we haven’t achieved our goal (group interview participant)

Overall, interviewees expressed that the actualization of KT and diffusion depends more on individual initiative than on a structured and organized system. Some participants also noted that these functions have become increasingly dynamic, moving away from a passive dissemination model, which has made them more complex and resource-intensive to implement.

To support these functions, participants suggested embedding KT into organizational structures, dedicating resources for knowledge brokering, reinforcing the responsibility of designated centers to support knowledge sharing across the system, recognizing KT as a driver of organizational learning, and developing mechanisms to enhance the visibility and recognition of research expertise.

### Leading practice function

Participants expressed significant confusion regarding this function, often using terms such as innovation, evidence-based practices, promising practices, cutting-edge practices, and leading practices interchangeably. One interviewee with expertise in leading practices provided the following clarification:

Often leading practices are the result of an innovation. But not every promising practice or innovation is, or become, a leading practice

Building on this definition, the function can be understood as a process that involves: (1) identifying or developing practices that address an unmet need and offer a significant improvement over existing practices; (2) evaluating these practices to assess their outcomes, and (3) promoting the scaling-up of leading practices to new contexts while ensuring their adaptability. This process calls for close integration of other academic functions, notably research, HITA, KT, and diffusion.

To strengthen the implementation of this function, participants highlighted the importance of providing support for local innovations, increasing the capacity of dedicated teams, clarifying the mandate and expectations of these teams to ensure consistency and rigor across institutions, and fostering active collaboration between centers to share promising practices and avoid duplication of efforts across the system.

## Discussion

In 1991, the province of Quebec (Canada) became one of the first jurisdictions to implement a government-led academic designation process for university-affiliated health and social service institutions. This process required the development of a national vision and designation criteria. In 2004, the Hendlisz report recommended revising this vision to align with the structural reform of that year. Following another reform in 2015, the MSSS recognized the need to update the vision of the academic mission and to review the designation process and criteria. Despite these calls for revision, this study suggests that the current vision has remains largely aligned with previous iterations, aiming to support care and services to improve population health and well-being.

This qualitative study contributes to this ongoing reflection by providing an in-depth perspective on how the academic mission is currently perceived within Quebec’s health and social services system. A revised vision for the academic mission should emphasize integration, collaboration, and impact measurement. Although coordination mechanisms exist, a more structured approach to managing the academic mission at local, regional, and national levels could enhance complementarity across functions. While some functions need further clarification, participants’ definitions suggest a shift toward a more integrated and collaborative vision. Recognizing KT, diffusion, and leading practices as integrative components could help AHCs move beyond the traditional tripartite mission, toward a more synergistic and operational model.

The purpose of the academic mission, as expressed by interviewees, aligns with recent international articles (Borden *et al.*, 2015; Gonzalo *et al.*, 2021; Grumbach *et al.*, 2014; King *et al.*, 2016; Pardes and Pincus, 2010; Raus *et al.*, 2020), which emphasize what Wartman (2010) calls “*the true mission [of AHCs] – improved health and well-being”*. However, while the international literature mainly focuses on defining and describing the three core functions, interviewees in this study formulated broader academic mission objectives that encompass all functions. These objectives reflect a growing interest in integrating academic functions, a process that involves bringing together actors from all functions to foster collaboration, define common goals, and develop a shared identity (Gonzalo *et al.*, 2021). This perspective is also consistent with the evolution of AHC models, which are increasingly structured as networks rather than traditional one-to-one relationships between a university and a health and social services center (Wartman, 2015). Integration and networking were also highlighted by participants as key components of the academic mission, consistent with evolving AHC models.

### Persistent challenges of the academic mission

The academic mission literature largely focuses on the challenges faced by AHCs. These challenges are consistent across different contexts and primarily relate to integration (Gonzalo *et al.*, 2021; Mann and Hess, 2015; Ovseiko *et al.*, 2014; Sanfilippo, 2010), funding and resources constraints (Cardinaal *et al.*, 2021; French *et al.*, 2014; Mann and Hess, 2015; Ovseiko *et al*., 2010, 2014; Raus *et al.*, 2020; Sanfilippo, 2010; Stimpson *et al.*, 2014) and the ongoing need to better define the mission and its functions (Cardinaal *et al.*, 2021; French *et al.*, 2014; Kubasik, 2017; Mann and Hess, 2015; Ovseiko *et al.*, 2014; Safarani *et al.*, 2018; Smith, 2009; Ward, 2002). As highlighted in the results, Quebec’s AHCs face similar obstacles, which have persisted over time (AQESSS, 2012).

Despite these challenges, professionals and managers perceive numerous benefits, a finding echoed in several articles (Borden *et al.*, 2015; Davies *et al.*, 2010; Edelman *et al.*, 2018; Ho *et al.*, 2014; Huang *et al.*, 2009; King *et al.*, 2016; Linegar *et al.*, 2012; Ovseiko *et al.*, 2015; Pardes and Pincus, 2010; Safarani *et al.*, 2018; Sanfilippo, 2010; Smitherman *et al.*, 2019). These included the advancement of care and service quality, the evolution of clinical practices, the development of recognized areas of expertise, increased opportunities for institutional recognition, and enhanced credibility. While these benefits are often reported with limited scientific support, mainly due to the difficulty of measuring long-term outcomes, participants nonetheless perceived them as central to their vision of the academic mission. They emphasized that such benefits outweighed the barriers, providing motivation to pursue academic activities despite ongoing challenges. For instance, integration and collaboration, though challenging, were also seen as key enablers, a perspective supported by international literature (French *et al.*, 2014; Gonzalo *et al.*, 2021; Grumbach *et al.*, 2014; King *et al.*, 2016; Raus *et al.*, 2020; Safarani *et al.*, 2018; Wartman, 2015).

### Defining and differentiating core academic functions

As mentioned earlier, naming specific academic functions remains a common strategy for defining the academic mission, as seen in participant responses despite the interview’s holistic approach. Unsurprisingly, research and education were the most frequently mentioned functions. These foundational academic functions engage professionals across designated and non-designated centers. According to the results, all health and social services centers participate in research and education activities, albeit at varying levels, while other academic functions are primarily carried out by designated centers. In contrast, specialized and highly specialized care and services was rarely mentioned as a function of the academic mission. Although this function is recognized as a core academic function in international literature, it also represents the primary mission of health and social services centers. Consistent with Wartman’s virtuous cycle (2008), which emphasizes that academic functions should be integrated to support care and services, most participants agreed that care and services should be supported by the academic mission. However, they did not necessarily perceive care and services as an academic function. Some interviewees did not associate specialized and highly specialized care and services exclusively with designated AHCs, questioning their relevance to the academic mission. A few participants even found it problematic that terms such as “high-quality care and services” or “excellence of care and services” were used only in reference to designated academic centers. As reflected in the results, participants preferred to define the care and services function in terms of area of expertise.

The definitions for the three classic functions (research, education, care and services) in this study align with international literature (Brown, 2018; French *et al.*, 2014; Pardes and Pincus, 2010; Sanfilippo, 2010) and government frameworks (Ministère de la Santé et des Services sociaux, 2013, 2010). However, this study refines these definitions. The proposed definition of research is closely tied to translational research, an approach widely promoted for AHCs (Borden *et al.*, 2015; Delaney *et al.*, 2010; French *et al.*, 2014; Raus *et al.*, 2020; Sanfilippo, 2010). Similarly, the education function includes both student training and continuous professional development. While the latter is less explicitly emphasized in the literature (Pardes and Pincus, 2010; Sanfilippo, 2010), perhaps because all centers are expected to promote ongoing training, AHCs may be expected to take a proactive role in its development and delivery. Interviewees also stressed that education should extend beyond clinical staff to include all professionals in health and social services centers (i.e. management, finance, human resources, logistics, etc.) (Brown, 2018; French *et al.*, 2014). Finally, specialized and highly specialized care and services were defined as focusing on a specific area of expertise in which other academic functions are integrated, rather than encompassing all care and services provided within a center. This perspective diverges somewhat from the ministry’s official definition (Ministère de la Santé et des Services sociaux, 2010) but aligns with Sanfilippo’s model (2010), which differentiates AHCs based on the “breadth and depth” of a specific healthcare specialty.

### Emerging and Quebec-specific functions

Some other academic mission functions identified in this study, while specific to Quebec, are increasingly recognized as essential components of the academic mission. KT and diffusion are particularly difficult to distinguish in the literature, with Sanfilippo (2010) even proposing that they are components of the education function. Regardless of the terms used, both the literature (Borden *et al.*, 2015; Edelman *et al.*, 2019; Gunashekar *et al.*, 2013; Raus *et al.*, 2020; Sanfilippo, 2010; VanLare *et al.*, 2011) and this study emphasize their critical role in the academic mission. KT and diffusion play a key role in the actualization and integration of the academic mission within AHCs (Côté-Boileau *et al.*, 2020). Most interviewees, in agreement with some reviewed articles, stressed that these functions must extend beyond scientific publication and communication (Borden *et al.*, 2015; Raus *et al.*, 2020; Sanfilippo, 2010; VanLare *et al.*, 2011). Instead, they should employ diverse strategies to influence decision-making, shape policy, and inform training programs, ultimately contributing to practices change and improved care and services.

The HITA function within the academic mission appears to be unique to Quebec’s health and social services system. While health technology assessment (HTA) is widely conducted internationally (International Network of Agencies for Health Technology Assessment (INAHTA), 2020), it is not typically recognized as an AHC function. Instead, it is often considered part of the research function, as it involves systematic literature review and evaluations, or as part of the KT function, given its role in informing decision-makers. Some authors have proposed expanding the academic mission to include functions like HITA. Sanfilippo (2010) suggested adding “healthcare technology creation, development, and application” as an academic function (Sanfilippo, 2010), while VanLare *et al.* (2011) (VanLare *et al.*, 2011) proposed “comparative effectiveness research”. Although these concepts share similarities with HITA, they do not fully align with the international definition of HTA (International Network of Agencies for Health Technology Assessment (INAHTA), 2020), the official MSSS definition, or the definitions provided by interviewees. Previous reports have noted challenges in structuring HITA, and this study confirms ongoing difficulties in defining its role and implementation. While HITA reports are recognized as useful, relatively few are effectively translated into practice. The current trend toward diversifying HITA outputs and strengthening collaboration with KT (McSween-Cadieux *et al.*, 2024; Hong *et al*., 2025) could help improve decision-making support and facilitate the integration of evidence into care and services, but further research is needed.

The leading practices function may correspond to the innovation function described in some articles (Borden *et al.*, 2015; King *et al.*, 2016; Sanfilippo, 2010). Many interviewees used both terms interchangeably, struggling to distinguish between them, a confusion also noted in different reports on Quebec’s academic mission (Duplantie, 2005; Hendlisz, 2004). However, innovation is generally considered a precursor to leading practices. Leading practices emerge from an innovative idea that must progress through phases of evaluation and research, followed by dissemination, KT, and scale-up, ultimately becoming evidence-based practices within a specialized area of expertise (Borden *et al.*, 2015; Hendlisz, 2004). This function relies on a collaborative, interdisciplinary structure, integrating multiple academic functions (Borden *et al.*, 2015; Hendlisz, 2004; King *et al.*, 2016), playing a key role in bridging the academic and care missions within health and social services centers.

### Towards a harmonized vision of the academic mission

This project highlights key areas for action to better structure and integrates the academic mission within the healthcare and social services system, positioning it as a driver of innovation and improvement:

1. Strengthening national positioning by recognizing the academic mission’s role in addressing current and future system challenges.
2. Enhancing integration mechanisms to promote collaboration, synergy, and knowledge-sharing across institutions.
3. Harmonizing designation criteria through a common framework that acknowledges institutional specificities and existing designations.
4. Clarifying the designation process and ensuring a flexible, well-understood renewal mechanism for all designations.
5. Providing institutional support (e.g. a dedicated ministerial team to assist institutions) and financial resources for academic mission activities.

### Study limitations

Group interviews provide access to a diverse range of participants, making them well-suited for decision-making research (Baribeau and Germain, 2010). However, some limitations must be considered. Social norms may influence participants’ responses (Baribeau and Germain, 2010). In a group setting, individuals may be inclined to conform to dominant ideas, and some participants may take up more space in the discussion, while others may feel less comfortable speaking. To mitigate this, interviewers actively encouraged balanced participation. Additionally, as with any interview technique, data can be influenced by the presence of researchers or other influential figures (Baribeau and Germain, 2010). To minimize this risk, the research team remained as neutral as possible, strictly adhering to the interview guidelines and clearly stating that all perspectives were valued equally. Finally, the large group size may have limited in-depth exploration of certain themes, though participants were invited to provide additional insights via email.

## Conclusions

This study offers a detailed account of how the academic mission is perceived within Quebec’s health and social services system. While the findings are embedded in a specific provincial context, they raise important questions for AHCs elsewhere. The results suggest that better recognition and integration of academic functions could promote innovation and continuous learning, enhance interdisciplinary collaboration, and foster the adoption of evidence-informed practices. A clearer and shared vision of the academic mission can help organizations better leverage these functions as strategic drivers for improving care and services. More broadly, this work contributes to ongoing discussions about the value and governance of AHCs and could inform the refinement or development of clearer frameworks or guidance related to the academic mission.

In addition, this study highlights the need for further comparative research across jurisdictions, as well as studies examining the impact of academic functions on care quality, service improvement, and health outcomes, as these areas remain underexplored. Ultimately, this study invites health system leaders to reimagine AHCs not as static structures but as dynamic platforms capable of evolving to better respond to population needs and advance quality in health and social services.

**List of abbreviations**
AHC: Academic Health Center
HITA: Health intervention and technology assessment
KT: Knowledge translation
MSSS: Ministère de la Santé et des Services sociaux (ministry of Health and Social Services)
CSSS: Centre de santé et services sociaux (health and social service centers)
CISSS: Centre intégré de santé et services sociaux (integrated health and social services centers)
CIUSSS: Centre intégré universitaire de santé et services sociaux (Integrated university health and social services centers)
RUISSS: Réseau universitaire intégré de santé et services sociaux (Integrated academic health and social services regional network)

## Supplementary Material

Data supplement 1

## Notes

The supplementary material for this article can be found online.
