# Supplementary material for: Perspectives on the academic mission in Quebec’s health and social services system: a qualitative study
Source: J Health Organ Manag. 2025 Aug 19;39(9):360–79. doi: 10.1108/JHOM-02-2025-0105 (PMC12492451; doi:10.1108/JHOM-02-2025-0105)
Supplement: Data supplement 1 [file jhom-02-2025-0105_suppl1.docx]

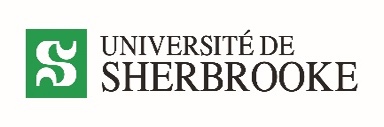


**SCIENTIFIC SUPPORT FOR UPDATING THE ACADEMIC MISSION AND ACADEMIC DESIGNATION CRITERIA OF INSTITUTIONS IN THE HEALTH AND SOCIAL SERVICES NETWORK**

**INTERVIEW GUIDE**

**To start, we would like to go around the table and ask each of you to briefly introduce yourselves:**

- Your position within your organization.
- Your role in the academic mission.

**We would like to discuss the academic mission of CIUSSS^^[[1]](#footnote-1)^^ and the meaning of the “U”. To begin, here are a few broad questions:**

- How do you define the academic mission? What does the “U” represent? What is its primary purpose?
- What should be the expected outcomes or impacts of the academic mission for the designated institution, the MSSS^^[[2]](#footnote-2)^^, the RSSS^^[[3]](#footnote-3)^^, and society as a whole?
- How can the academic mission be integrated both vertically (across different levels of the institution) and horizontally (across departments and services)? (e.g., What governance and management structures should support the academic mission?)

**Next, we would like to explore the functions of the academic mission.**

- **First, we will examine the four core functions of the academic mission in Quebec: research, education, care and services, and Health Intervention and Technology Assessment^^[[4]](#footnote-4)^^ (HITA). We will discuss each function separately.**
  - i. How important is [name of the function] in your view?
  - ii. How does this function contribute to achieving the institution's academic mission?
  - iii. How should this function be implemented and integrated within the institution?
  - iv. What indicators could be used to monitor the deployment of this function and assess its impact?
- **We will now turn to additional functions that have been introduced over the years, namely knowledge transfer and diffusion, as well as leading practices. Again, we will discuss each function separately.**
  - i. How important is [name of the function] in your view?
  - ii. How does this function contribute to achieving the institution's academic mission?
  - iii. How should this function be implemented and integrated within the institution?
  - iv. What indicators could be used to monitor the deployment of this function and assess its impact?

Before concluding, is there anything you would like to add that you consider essential for updating the academic mission and designation criteria of institutions in the health and social services network?

Thank you for your participation.

1. Integrated health and social services academic centre [↑](#footnote-ref-1)
2. Health and social services ministry [↑](#footnote-ref-2)
3. Health and social services network [↑](#footnote-ref-3)
4. Health interventions and technologies assessment [↑](#footnote-ref-4)
